# Design and outcome measures of clinical trials for active ulcerative colitis: a scoping review based on databases

**DOI:** 10.3389/fimmu.2026.1932604

**Published:** 2026-10-19

**Authors:** Xin Zhao, Changzhuo Feng, Chao Zhao, Runfeng Zhang, Shuo Zhang, Ran Wang, Jiahui Guo, Chengqiu Yan, Liang Chen

**Affiliations:** 1 College of Traditional Chinese Medicine, Changchun University of Chinese Medicine, Changchun, China; 2 The Affiliated Hospital to Changchun University of Chinese Medicine, Changchun University of Chinese Medicine, Changchun, China

**Keywords:** colitis gravis, idiopathic proctocolitis, inflammatory bowel disease, ulcerative colitis, ulcerative colitis type

## Abstract

**Background:**

Ulcerative colitis (UC) reduces the quality of life for patients and creates a significant economic burden on global healthcare systems. Although there have been advances in UC clinical trials recently, most existing reviews have primarily focused on pathogenesis or pharmacological treatments. This review analyzed the characteristics of current clinical trials for active ulcerative colitis and assessed research methods. This review focused on refining trial design and providing evidence-based methods for improving treatment outcomes and prognosis.

**Method:**

This scoping review was conducted in accordance with the PRISMA extension for Scoping Reviews (PRISMA-ScR). The study searched clinical trials registered in ClinicalTrials.gov and the Chinese Clinical Trial Registry (ChiCTR) from April 1, 2016, to April 1, 2026. In addition, the study searched PubMed for clinical trials published between April 1, 2016, and April 1, 2026. Data were charted and presented in tables.

**Result:**

The study revealed a rapid increase in the number of clinical trials focusing on active ulcerative colitis since 2019. Biologics and small-molecule targeted drugs have become the core interventions, while traditional therapies constitute a smaller proportion of all treatments. However, these studies face limitations, such as small sample sizes, short follow-up periods, and an imbalance in outcome measures, which heavily rely on the Mayo score while underutilizing patient-reported outcomes and novel endoscopic scoring tools.

**Conclusion:**

The number of clinical trials for active ulcerative colitis has increased, and intervention strategies have become more varied. However, there is significant variation in study populations, trial design, and the evaluation of their effectiveness. Future trials should prioritize large-scale, long-term studies with comprehensive and patient-centered outcome measures.

## 1 Introduction

Ulcerative colitis (UC) is a common disease within the field of gastroenterology, classified as a chronic inflammatory bowel disease (IBD). The lesions of UC are confined to the mucosal and submucosal layers of the colon, affecting a portion or the entire colon. Typical clinical manifestations of UC include urgent bowel movements, mucus and bloody purulent stools, abdominal pain, and fatigue (1). Throughout the course of UC, periods of remission and active disease often alternate (2). A systematic review examining global trends in the hospitalization of UC patients revealed that since 2000, Western countries experiencing a compounding prevalence have reported an incidence rate exceeding 0.5%, accompanied by a stable trend in hospitalization rates. In contrast, newly industrialized countries, particularly in Asia and Latin America, exhibit a lower incidence of UC but are witnessing a rapid increase in cases, accompanied by a significant rise in hospitalization rates (3). The pathogenesis of UC remains incompletely understood. Current research indicates that the onset of UC is associated with genetic factors, immune dysregulation, environmental and psychological influences, and disruptions in gut microbiota (4–10). Due to the long-term chronic inflammatory stimulation associated with UC, colitis-associated colorectal cancer is the most serious complication of UC (11). Therefore, early management of UC and its treatment are particularly important, which can be achieved through the use of steroids, immunomodulators, and biologics to control and alleviate the disease (12). Clinical trials play a crucial role in assessing the efficacy of new therapies and refining existing treatment protocols. The rigor and quality of clinical trial design decisively impact the scientific validity and effectiveness of research outcomes (13).

This review utilized ClinicalTrials.gov, Chinese Clinical Trial Registry (ChiCTR), and PubMed as data sources. ClinicalTrials.gov is the largest global database for the registration and results reporting of clinical trials, maintained by the U.S. National Library of Medicine. It consolidates data on ongoing and completed studies worldwide (14). Since its establishment in June 2007, ChiCTR has joined the World Health Organization’s International Clinical Trial Registry Platform(WHO ICTRP) as a registered institution (15). As the most comprehensive biomedical literature database, PubMed significantly aids global researchers in conducting systematic reviews (16).

Current literature encompasses comprehensive reviews addressing the pathogenesis, therapeutic strategies, and dietary interventions associated with UC. In recent years, network pharmacology approaches have been extensively utilized to investigate the role of gut microbiota metabolites in ulcerative colitis. These studies have uncovered potential mechanisms through which therapeutic effects are exerted via multiple targets and pathways (17). However, there is a notable lack of scoping reviews concerning the design and outcomes of clinical trials related to ulcerative colitis.Moreover, heterogeneity in outcome measures, trial design, and disease severity across clinical trials has hindered the synthesis and dissemination of research findings (18–20). This study aims to conduct a scoping review of the current landscape of clinical trial design for active ulcerative colitis, based on the data from ClinicalTrials, ChiCTR, and PubMed. Therefore, it is necessary to comprehensively map and analyze the current clinical trial designs in order to provide better guidance for optimizing future trial protocols and selecting appropriate endpoints.

## 2 Methods

### 2.1 Search strategy

This scoping review was conducted following the methodological framework originally proposed by Arksey and O’Malley and further refined by the Joanna Briggs Institute (JBI). The reporting of this review followed the Preferred Reporting Items for Systematic Reviews and Meta-Analyses extension for Scoping Reviews (PRISMA-ScR) checklist (21, 22).

The search covered records registered or published between April 1, 2016, and April 1, 2026 in ClinicalTrials.gov, ChiCTR, and PubMed. The 10-year period from April 1, 2016, to April 1, 2026, was selected to capture the most recent decade of clinical trial activity in active UC. This timeframe was also chosen to reflect current trial design and outcome measure practices, while limiting heterogeneity from older trials. The researchers (Xin Zhao and Changzhuo Feng) conducted a literature search using a search strategy that combined controlled vocabulary and free-text terms related to ulcerative colitis. The search strategies were tailored to the specific interface and indexing system of each database. For PubMed, free-text terms were used as follows: (Colitis Gravis[Title/Abstract] OR Idiopathic Proctocolitis[Title/Abstract] OR Inflammatory Bowel Disease[Title/Abstract] OR Ulcerative Colitis Type[Title/Abstract] OR Ulcerative Colitis[Title/Abstract]), with the article type filters “Clinical Trial” and “Randomized Controlled Trial” applied. For ClinicalTrials.gov, the search was conducted using the “Condition or disease” field with the terms “Colitis Gravis”, “Idiopathic Proctocolitis”, “Inflammatory Bowel Disease”, “Ulcerative Colitis Type” and “Ulcerative Colitis” combined with the “Study type: Interventional” filter. For ChiCTR, the search was performed using the Chinese equivalents “Colitis Gravis”, “Idiopathic Proctocolitis”, “Inflammatory Bowel Disease”, “Ulcerative Colitis Type” and “Ulcerative Colitis”, as well as their English translations, within the “Disease name” and “Title” fields. The full search strings for each database are provided in **Supplementary Table 2**. No language restrictions were applied. Grey literature, conference abstracts, and unpublished data were not searched.

### 2.2 Inclusion and exclusion criteria

The inclusion criteria were as follows: 1) Study population: patients diagnosed with active UC, including mild, moderate, and severe cases; 2) Intervention: any intervention evaluating adjunctive therapy as the main treatment for active UC, including pharmacological therapy, gut microbiota modulation therapy, dietary intervention, hyperbaric oxygen therapy, nutritional supplements, cell therapy, and ethnic medicine treatment; 3) Study type: interventional clinical trials, including randomized controlled trials, non-randomized controlled trials, and single-arm trials; 4) Time frame: studies indexed in PubMed, limited to the past 10 years, with the publication date restricted to after April 1, 2016.

Exclusion criteria were as follows: 1) Studies unrelated to UC (e.g., Crohn’s disease or IBD without UC-specific data); 2) Surgical treatment approaches (e.g., ileal pouch-anal anastomosis); 3) Non-clinical studies (e.g., basic research, reviews, observational studies without intervention); 4) Studies for which full text or sufficient data could not be obtained.

The inclusion of non-randomized and single-arm trials was intentional, as this scoping review aimed to capture the full spectrum of interventional trial designs for active UC, including early-phase and exploratory studies that are often excluded from systematic reviews focusing on comparative effectiveness.

### 2.3 Date charting

A standardized data charting form was developed in Microsoft Excel through an iterative process. We used Excel to develop a standardized data charting form covering the trial name and registration number, study population, UC severity, participant characteristics, study design, primary and secondary outcome measures, country, and other variables. Two reviewers (Chao Zhao and Runfeng Zhang) first performed a pilot extraction of the data to calibrate the rules and optimize the content, thereby ensuring consistency and feasibility. For trials registered on ClinicalTrials.gov, and in ChiCTR, and indexed in PubMed, duplicates were identified by matching the trial title, principal investigator and sponsoring institution. When a repetitive trial appeared in registries, the record with more complete information was retained, and the other was excluded as a duplicate. For trials identified in PubMed that corresponded to a registered trial already identified in ClinicalTrials.gov or ChiCTR, the published article and the registry record were linked using the registration number reported in the publication, or by matching the trial title, intervention, sample size, and study period when the registration number was not reported. In such cases, the registry record and the publication were treated as a single study, and data were charted from both sources to maximize completeness. They then independently extracted all eligible data and cross-checked the results. For any discrepancies, they re-reviewed the original trials and discussed the findings until consensus was reached; if consensus still could not be achieved, a third senior investigator (Liang Chen) made the final decision, which was adopted. Each clinical trial was counted only once in the final analysis. The 522 included studies therefore represent 522 unique clinical trials rather than 522 separate records.

### 2.4 Statistical analysis

Data were analyzed using Microsoft Excel. To align with the descriptive and mapping-oriented objective of this scoping review, the extracted data were summarized using descriptive statistics, including frequencies and percentages. Results are presented in tabular and graphical formats, including tables of study characteristics, intervention classification, outcome measures, and trial design, as well as figures illustrating temporal trends, geographic distribution, intervention categories, and sample size distribution. No meta-analysis or quantitative evidence synthesis was performed.

### 2.5 Critical appraisal

Consistent with the methodological framework for scoping reviews, no formal critical appraisal or risk of bias assessment was performed (21). Scoping reviews are designed to map the existing evidence and identify research gaps, rather than to assess the quality of individual studies or synthesize effect estimates. Therefore, the findings of this review should be interpreted as a descriptive overview of the current landscape of clinical trial design for active UC, not as a quality-graded synthesis of evidence.

## 3 Results

### 3.1 Study selection and inclusion

This study ultimately included 522 clinical trials. A total of 2821 records were retrieved from the ClinicalTrials, ChiCTR, and PubMed databases. After removing duplicates, 54 records were excluded. The remaining 2767 records underwent full-text screening. A total of 2245 trial studies that did not meet the inclusion criteria were excluded, including studies on Crohn’s disease and those focused on exploratory diagnostic indicators. Finally, 522 clinical trials were included in this study. The literature screening process is shown in **Figure 1**.

**Figure 1:**
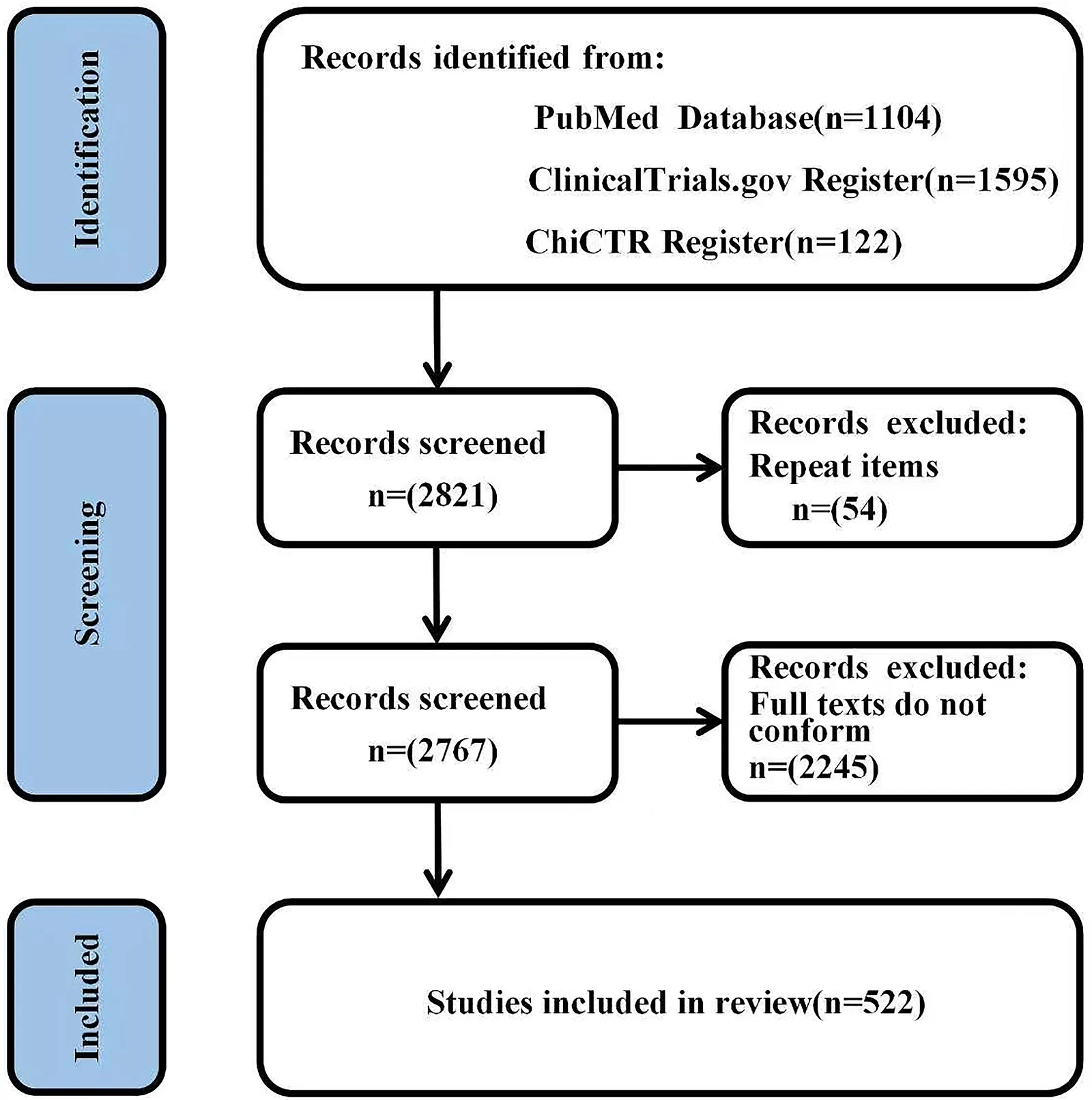
PRISMA search flowchart.

### 3.2 Number of registrations and publications, country

The overall number of registrations and publications showed a gradual upward trend over time. Since 2019, the field has entered a phase of rapid expansion, with a substantial increase in the number of studies. The largest increase occurred from 2018 (37 studies) to 2019 (55 studies), reaching 48.65%. This suggests that clinical trial research on active UC has become more active since 2019. Countries in Europe (Germany), Asia (Iran), and Africa (Egypt) were the most prominent. There were no related clinical trials before 2019; research on active UC began to emerge from 2019 onward. Starting in 2019, the number of registrations entered a high-level plateau, with most years recording more than 50 registrations. The number of registrations was highest in 2023, with 65 registrations. Although there was a slight decline afterward, the annual number remained above 50 each year. However, the number of registrations in 2026 was 21. This is likely because the inclusion period for 2026 only covered studies registered before April 1, 2026, rather than the full year.

From the perspective of the countries of publication or registration, the United States (182 studies, 34.87%) and China (111 studies, 21.26%) dominated the research trials on active ulcerative colitis, with the combined total from both countries accounting for 56.13% of all included trials. In addition, Egypt had no related studies before 2021, but since then the number of research trials has increased rapidly, reaching a peak of 11 trials in 2023. The 26 studies registered in Egypt accounted for 4.98% of the total, making it the third most prolific country. However, some studies did not clearly specify the country in which they were conducted (25 studies, 4.79%). Austria, Sweden, Belgium, Italy, the United Kingdom, the Netherlands, and Germany all exhibited a notable level of activity in clinical trials of active UC, with 5–9 studies each. Denmark, France and other European countries, as well as Israel in the Middle East and India and Japan in Asia, have conducted a relatively large number of trials (9–18 studies). Over the past decade, their research activity has remained stable, making a consistent contribution to the understanding of the disease. **Figure 2** showed the number of registrations and published clinical studies in different years. **Figure 3** showed the number of registrations and published clinical studies in different countries.

**Figure 2:**
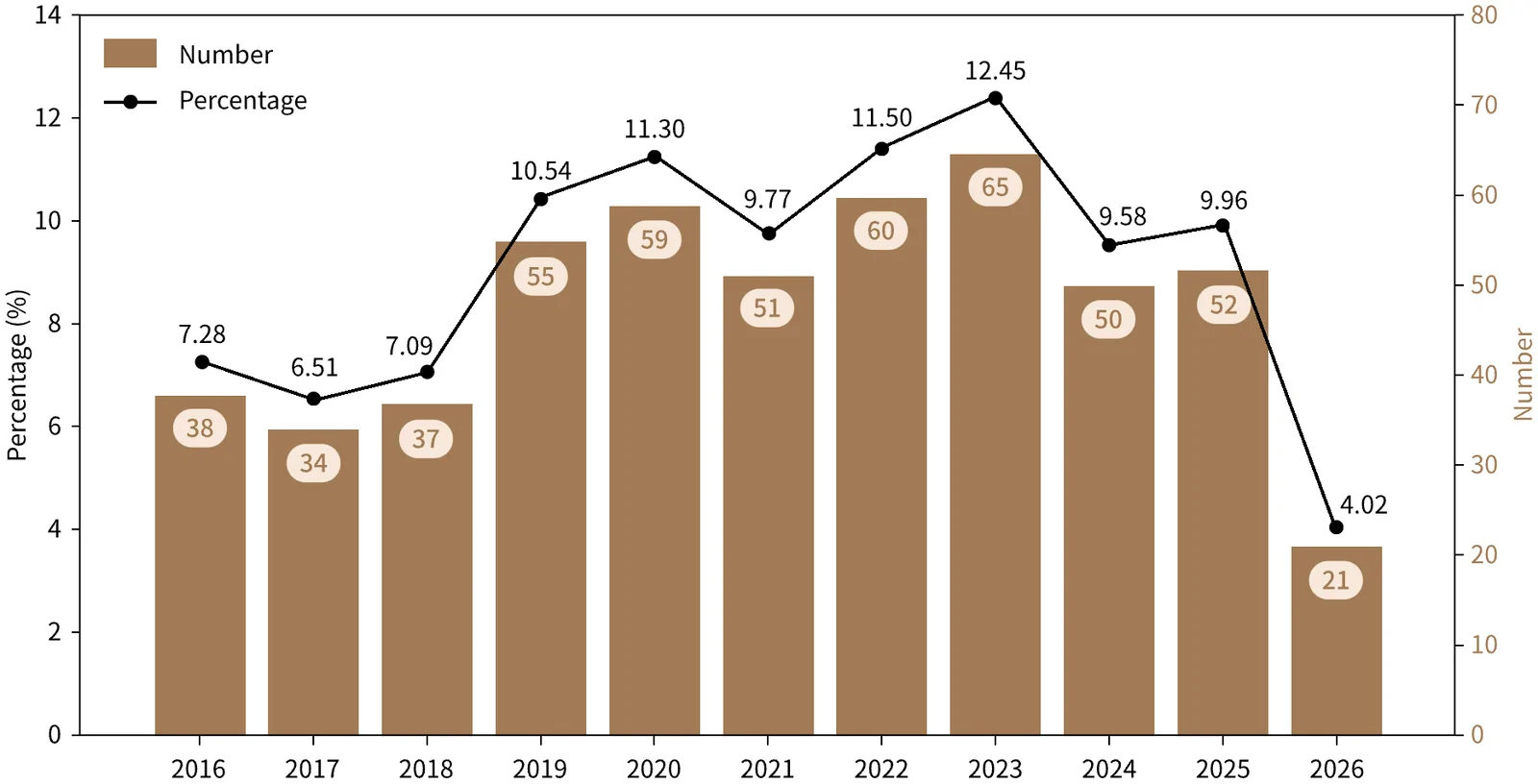
Bar and line chart combination depicting yearly trends from 2016 to 2026, with brown bars representing quantity and a black line with dots representing proportion. Both variables peak in 2023 and then experience a significant decline.

**Figure 3:**
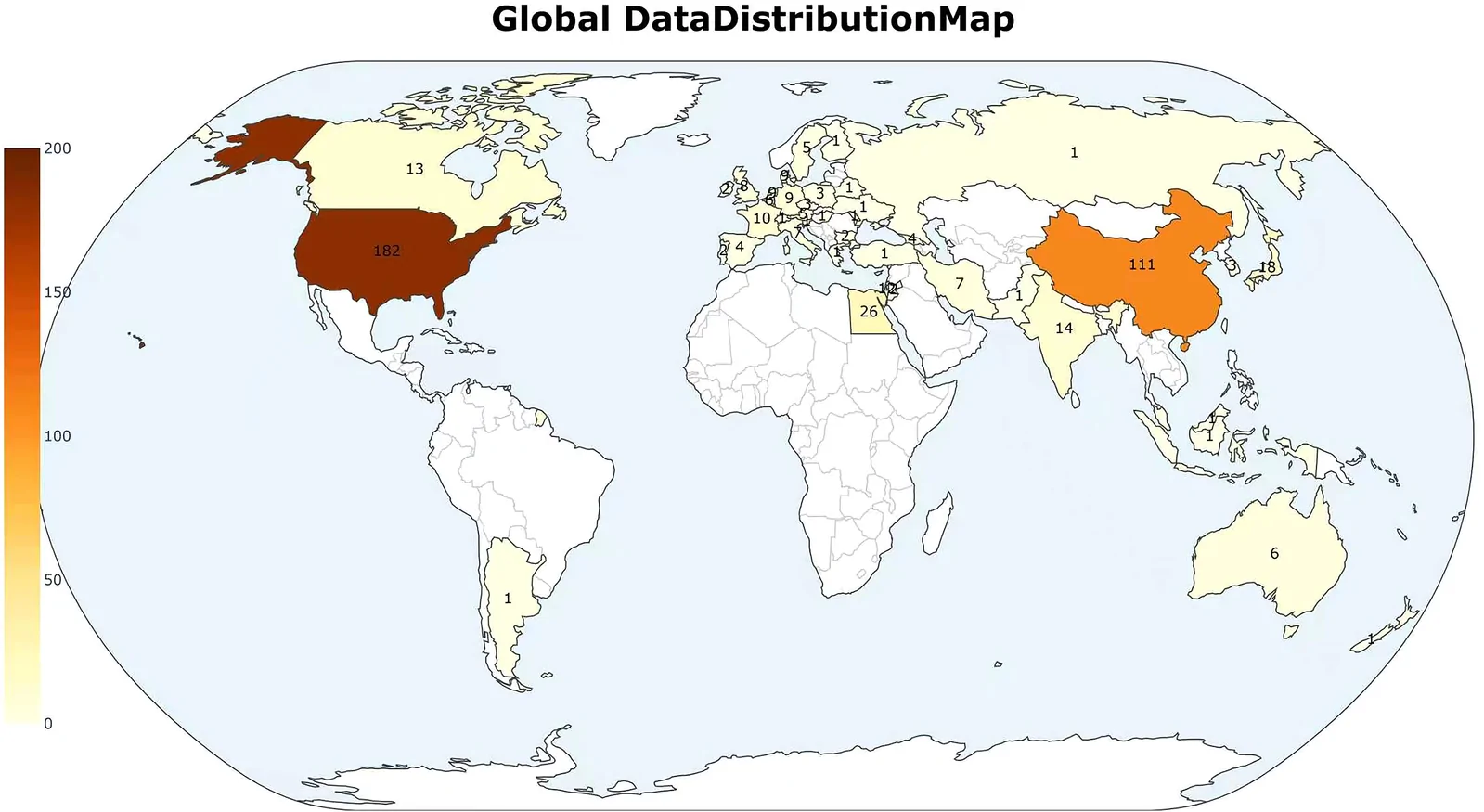
Published or registered country.

### 3.3 Characteristics of intervention

For the purpose of this review, “natural products and traditional Chinese medicine” refers to plant-, mineral-, or animal-derived preparations and Traditional Chinese Medicine formulae; “nursing interventions” refers to structured nursing care, patient education, and symptom management programs; and “dietary interventions” refers to specified dietary patterns or nutritional supplementation regimens.

Overall, the intervention characteristics can be divided into 16 major categories. Among these categories, biologics were the most common intervention type (116 studies, 21.25%). These were followed by small-molecule targeted drugs (77 studies, 14.10%), microbiome modulation and Fecal Microbiota Transplantation (FMT) (71 studies, 13.00%), natural products and traditional Chinese medicine (56 studies, 10.26%), which also accounted for a notable proportion of the trials. By contrast, relatively few trials investigated conventional therapeutic drugs, namely aminosalicylates, corticosteroids, and immunosuppressants (32 studies, 5.86%).

Among all interventions, several emerging approaches were identified in relatively small numbers, including various dietary supplements (33 studies, 6.04%), nursing interventions (23 studies, 4.21%), cellular and blood-derived interventions (17 studies, 3.11%), neurostimulation therapy (6 studies, 1.10%), and hyperbaric oxygen therapy (5 studies, 0.92%).

Among the intervention projects, some studies evaluated the clinical efficacy of drugs originally used for other diseases in patients with active UC. These included nutritional/metabolic/cardiovascular modulators (14 studies, 2.56%), antihistamines/antitussives/other chemical drugs (7 studies, 1.28%), drugs for liver disease (2 studies, 0.37%), and xanthine oxidase inhibitors (1 study, 0.18%). The intervention characteristics are shown in **Figure 4**.

**Figure 4:**
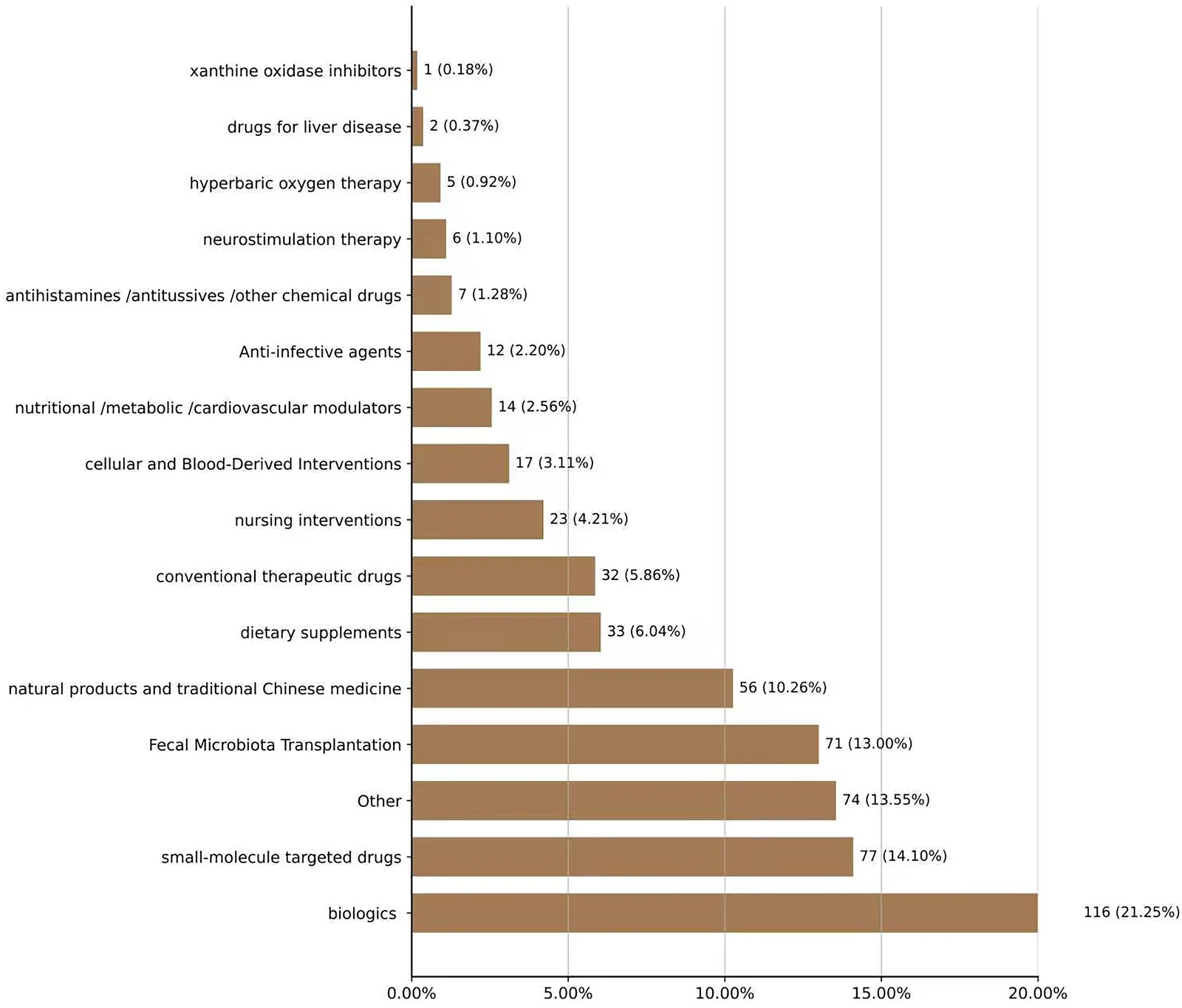
Classification of interventions.

When the two most frequently studied intervention categories were examined by year, biologics showed a fluctuating but generally sustained presence across the study period, with a notable increase in 2025 (16 studies). In contrast, FMT showed a gradual decline after 2022, decreasing from 11 studies in 2022 to 3 studies in 2026. These observations are limited to the two most frequently studied intervention categories and are descriptive in nature; no formal trend test was performed. The annual distribution of these two intervention categories is shown in **Figure 5**.

**Figure 5:**
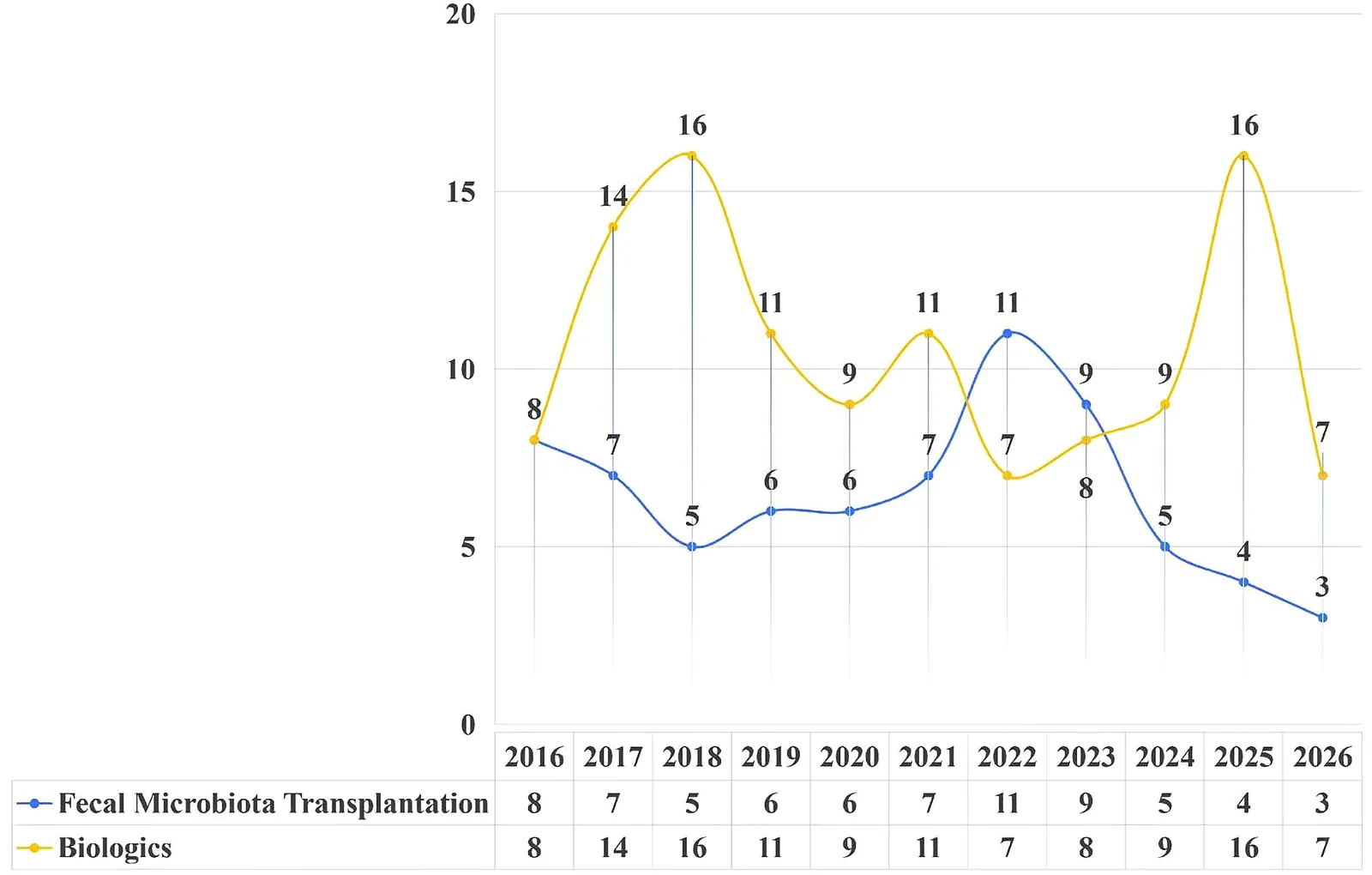
The annual distribution of fecal microbiota transplantation and biologics from April 1, 2016 to April 1, 2026.

### 3.4 Basic characteristics of the included population

#### 3.4.1 Basic characteristics of the inclusion criteria

In all included trial studies, we restricted the inclusion criteria to active UC. Among all 522 included trial studies, the Mayo score system was the most frequently used criterion for assessing disease activity (274 studies, 52.49%). Further analysis showed that, according to the reference subscore used, the Mayo score system consisted of the Full Mayo Score (FMS)(141 studies, 27.01%), the Modified Mayo Score (MMS)(115 studies, 22.03%), and the Partial Mayo Score (PMS)(18 studies, 3.45%). The FMS consists of four sub-scores: stool frequency score (SFS), rectal bleeding score (RBS), Mayo endoscopic score (MES), and physician global assessment (PGA). The MMS removes PGA from the FMS, while the PMS removes MES from the FMS. Regarding the distribution of the four sub-scores, we further counted the number of studies that specified particular score cutoffs for each of the four sub-scores in the inclusion criteria, MES was the most frequently specified score item in isolation (220 studies, 42.15%), followed by RBS (92 studies, 17.62%), SFS (48 studies, 9.20%), and PGA (7 studies, 1.34%). The characteristics of the inclusion criteria were summarized in **Table 1**.

**Table 1:** Characteristics of intervention population.

Intervened group	Included literature	Record percentage (%)
Mayo score system	274	52.49
Full Mayo score	141	27.01
Modified Mayo score	115	22.03
Partial Mayo score	18	3.45
Mayo endoscopic score	220	42.15
Stool frequency score	48	9.20
Rectal bleeding score	92	17.62
Physician global assessment	7	1.34
Moderate-to-severe ulcerative colitis	240	45.98
Mild-to-moderate ulcerative colitis	180	34.48
Moderate ulcerative colitis	30	5.75
Severe ulcerative colitis	21	4.02
Mild-to-severe ulcerative colitis	18	3.45
Mild ulcerative colitis	8	1.53
No restriction on severity	25	4.79

#### 3.4.2 Disease characteristics of the included sample

This study enrolled patients with active UC, which can be classified as mild, moderate, and severe. Among the disease severity categories in the trial studies, moderate-to-severe was the most common requirement (240 studies, 45.98%), followed by mild-to-moderate (180 studies, 34.48%); the others were moderate (30 studies, 5.75%), severe (21 studies, 4.02%), mild-to-severe (18 studies, 3.45%), and mild (8 studies, 1.53%). In addition, 25 studies (4.79%) did not clearly specify the severity of active ulcerative colitis, reflecting a lack of rigor in study design. The characteristics of the included sample were summarized in **Table 1**.

#### 3.4.3 Age and sexuality characteristics of the included sample

By analyzing the age eligibility criteria of 522 studies, we found that “18 years and older” was the most common age inclusion criterion (420 studies, 80.46%), while 11 trial studies did not specify any age restriction for participants. The remaining age cutoffs were distributed more sparsely. Accordingly, studies targeting adults and older adults also accounted for the vast majority (413 studies, 79.12%). In addition, 42 studies (8.05%) included “children, adults, and older adults” as their target population. The remaining population categories were adults (21 studies, 4.02%), children and adults (20 studies, 3.83%), children (19 studies, 3.64%), and unspecified (7 studies, 1.34%). This distribution suggests that participant recruitment in current clinical trials on active UC is highly concentrated in middle-aged populations, while studies involving children and older adults remain relatively scarce. The gender requirements in these studies are comparatively comprehensive. Most studies (520, 99.617%) did not restrict participant sex. In addition, one study (0.19%) limited participants to females, and one study (0.19%) did not clearly specify sex eligibility requirements. Age and Sexuality characteristics were summarized in **Table 2**.

**Table 2:** Intervention characteristics of active ulcerative colitis research.

Characteristics	Category	Included literature	Record percentage (%)
Sample size	0-50	207	39.66
	51-100	133	25.48
	101-150	67	12.84
	151-200	26	4.98
	201-250	29	5.56
	251-300	10	1.92
	301-350	8	1.53
	351-400	8	1.53
	401-450	4	0.77
	Over 451	30	5.75
Age	younger than 18 years	76	14.56
	18 years and older	420	80.46
	19 years and older	4	0.77
	20 years and older	6	1.15
	21 years and older	1	0.19
	35 years and older	1	0.19
	younger than 75 years	1	0.19
	18 years and younger	2	0.38
	NA	11	2.11
Sexuality	All	520	99.62
	Female	1	0.19
	Not specified	1	0.19
Experimental intervention time	1–7 days	27	5.17
	8 days-4 weeks (excluding 4 weeks)	20	3.83
	4 weeks-8 weeks (excluding 8 weeks)	65	12.45
	8 weeks-12 weeks (excluding 12 weeks)	108	20.69
	12 weeks-24 weeks (excluding 24 weeks)	136	26.05
	24 weeks and above.	117	22.41
	12 weeks	96	18.39
	Not specified	49	9.39
Follow-up time	1 month and within 1 month	53	10.15
	3 months and less than 3 months	55	10.54
	3–12 months (including 12 months)	87	16.67
	Over 12 months	22	4.21
	Not specified	305	58.43
Study population	Child	19	3.64
	Adult	21	4.02
	Child, Adult	20	3.83
	Adult, Older Adult	413	79.12
	Child, Adult, Older Adult	42	8.05
	NA	7	1.34

### 3.5 Basic characteristics of the studies

#### 3.5.1 Characteristics of the number of participants included

Among the 522 studies, the participant sample sizes were mainly concentrated in the 0–50 range (207 studies, 39.66%), the 51–100 range (133 studies, 25.48%), and the 101–150 range (67 studies, 12.84%). Smaller-scale studies were conducted more frequently, whereas trials enrolling more than 300 participants were relatively uncommon, accounting for only 50 studies (9.58%) and representing a sparsely distributed subset. These findings indicate that, under conditions of adequate evidentiary rigor and objective feasibility, studies with fewer than 100 participants can still provide meaningful reference value for this disease. Characteristics of the enrolled sample size are presented in **Table 2**. **Figure 6** illustrates the quantitative distribution characteristics across different sample size intervals.

**Figure 6:**
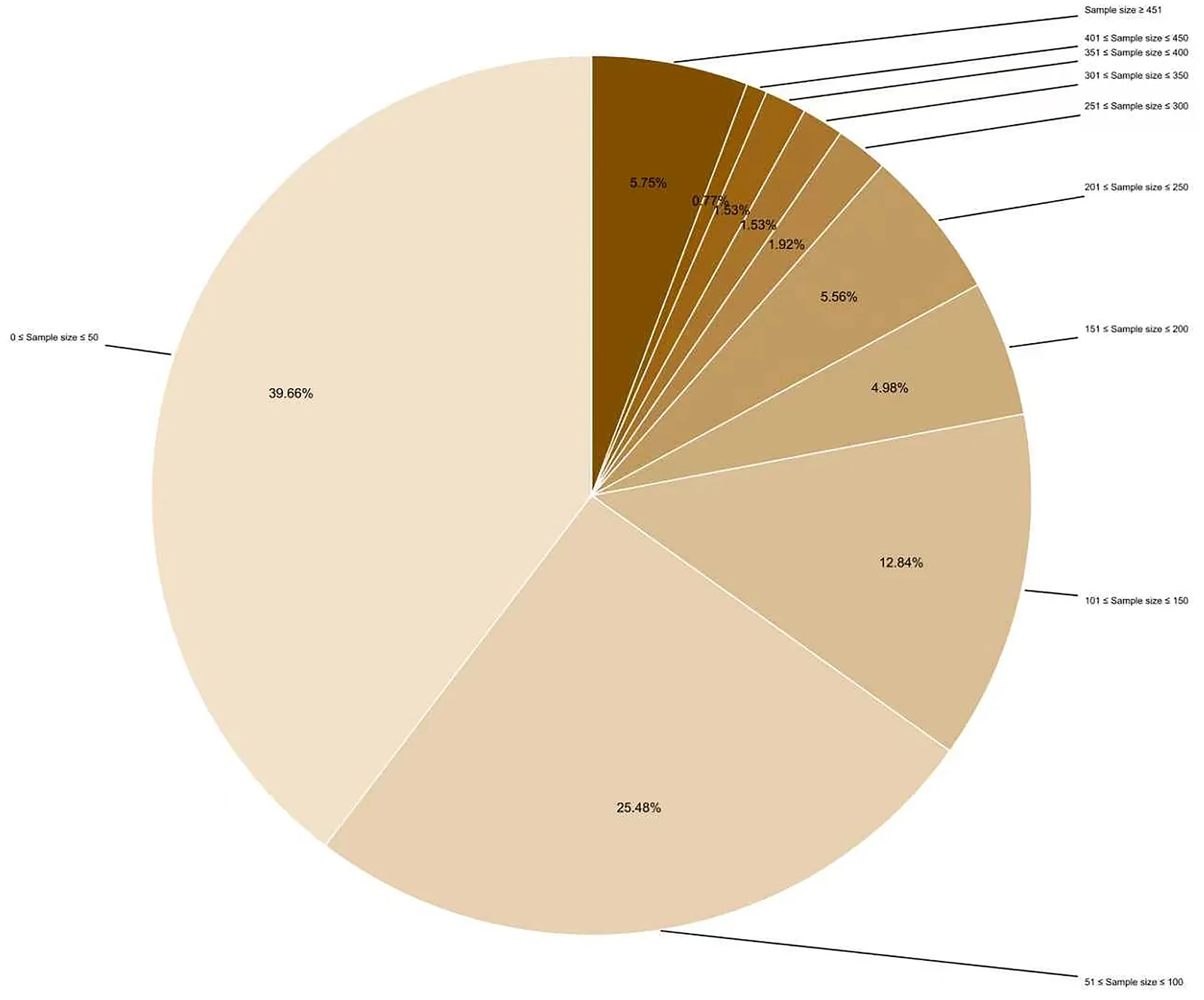
Illustrates the quantitative distribution characteristics across different sample size intervals.

#### 3.5.2 Intervention duration and trial design characteristics

Randomized controlled trials represent the principal clinical research design employed in studies of active UC. In terms of allocation methods, randomization predominated (420 studies, 80.46%), whereas nonrandomized allocation accounted for 18.58%. Regarding intervention design, parallel-group trials were the most common (419 studies, 80.27%), followed by single-arm studies (81 studies, 15.52%).

In terms of blinding design, open-label studies were the most common (156 studies, 29.89%), followed by double-blind studies (151 studies, 28.93%). The remaining trial design categories included quadruple-blind studies (100 studies, 19.16%), triple-blind studies (53 studies, 10.15%), single-blind studies (41 studies, 7.85%), and 21 studies that did not clearly report blinding design (4.02%).

Regarding the number of trial arms, two-arm designs were overwhelmingly dominant (277 studies, 53.07%), followed by three-arm designs (93 studies, 17.82%) and single-arm designs (81 studies, 15.52%). Complex multi-arm trials (four arms or more) comprised 71 studies, accounting for 13.60%. The study design of clinical trials was shown in **Table 3**.

**Table 3:** Research design of active ulcerative colitis.

Characteristics	Category	Included literature	Record percentage (%)
Allocation	Randomized	420	80.46
	Non-randomised	97	18.58
	Queue	1	0.19
	Case-Control	2	0.38
	NA	2	0.38
Intervention model	Parallel	419	80.27
	Single Component	81	15.52
	Factorial Assignment	2	0.38
	Cross-over	7	1.34
	Sequential Assignment	13	2.49
Masking/Blinding	Single blind	41	7.85
	Double blind	151	28.93
	Triple blind	53	10.15
	Quadruple blind	100	19.16
	Open Label	156	29.89
	NA	21	4.02
Set the number of test groups	Single-arm test	81	15.52
	Two arms test	277	53.07
	Three-arm test	93	17.82
	Four-arm test	50	9.58
	Five arms test	11	2.11
	Six-arm or more arms test	10	1.92

With regard to intervention duration, the included studies covered a relatively wide span, ranging from a few days to more than 24 weeks. Specifically, 220 studies (42.15%) had intervention periods of less than 12 weeks (excluding 12 weeks), 253 studies (48.47%) had intervention periods of 12 weeks or longer, and 49 studies (9.39%) did not clearly report the intervention duration. Among the studies that reported duration explicitly, those lasting 12–24 weeks (136 studies, 26.05%) and 24 weeks or longer (117 studies, 22.41%) were the most common, suggesting that longer-term intervention protocols were more frequently used; meanwhile, studies lasting 8–12 weeks (108 studies, 20.69%) and 4–8 weeks (65 studies, 12.45%) also accounted for a considerable proportion, indicating that medium-length intervention periods also received attention. In contrast, only 47 studies (9.00%) had intervention durations shorter than 4 weeks, representing a relatively small share. Overall, the distribution of intervention duration showed a clear tendency toward longer-term designs, reflecting a general recognition that intervention effects in this field require a certain amount of time to accumulate before they can be adequately evaluated. It also suggests that future studies should place greater emphasis on standardized reporting of intervention duration to facilitate cross-study comparison and evidence synthesis.

As many as 305 studies (58.43%) did not clearly report the follow-up period. Among the studies that did report follow-up, short-term follow-up within 3 months (including 3 months) was most common (55 studies, 10.54%), followed by medium-term follow-up of 3–12 months (including 12 months) (87 studies, 16.67%). Long-term follow-up of more than 12 months was reported in only 22 studies (4.21%). The characteristics of the clinical trials are shown in **Table 2**.

### 3.6 Basic characteristics of outcomes

In the following sections, percentages for individual outcome measures are calculated based on the number of trials within the corresponding subcategory, unless otherwise stated.

#### 3.6.1 Primary outcome measures

The primary outcome measures were characterized predominantly by the Mayo scoring system. The Mayo scoring system was used not only as an inclusion criterion to restrict the severity of active UC, but also as the primary outcome measure for evaluating post-intervention effects. The Mayo scoring system occupied a dominant position among the study outcome measures. In the primary outcome measures, the three Mayo scoring systems appeared a total of 246 times, accounting for 25.76% of the total. Among them, FMS and MMS were the most frequently used, appearing 107 and 116 times, respectively, whereas PMS appeared only 23 times. Analysis of the subitems within the Mayo score showed that some trials specified criteria for particular components. Among these, MES had the highest number of requirements (218 studies, 22.83%), followed by RBS (188 studies, 19.69%) and SFS (176 studies, 18.43%). In contrast, PGA was required least frequently (60 studies, 6.28%). A total of 33 trials used the Simple Clinical Colitis Activity Index (SCCAI) as the primary outcome measure, accounting for 3.46% of all studies. In addition, 13 trials used the Pediatric Ulcerative Colitis Activity Index (PUCAI) as the primary outcome measure, representing 1.36%. As a disease-specific assessment tool for pediatric ulcerative colitis, PUCAI was used relatively infrequently in studies involving this population, likely because the overall number of pediatric UC clinical trials was small.

A total of 117 clinical trials used physical examination and laboratory tests as the primary outcome measures, accounting for 22.41% of all trials. Fecal calprotectin (21 studies, 17.95%), C-reactive protein (15 studies, 12.82%), erythrocyte sedimentation rate (7 studies, 5.98%), and Immune-and Inflammation-Related Serological Markers (13 studies, 11.11%) were the most frequently assessed tests, forming an inflammatory evaluation system ranging from local intestinal inflammation to systemic inflammation. In addition, pharmacokinetic and metabolic assessments (19 studies, 16.24%) also accounted for a certain proportion of the overall research. Trials using safety and adverse events as the primary outcome measure were relatively numerous, with 96 studies. Characteristics of the primary outcome measures are presented in **Table 4**.

**Table 4:** Measurement of the results of active ulcerative colitis research.

Characteristics (Main evaluation index)	Category	Included literature	Record percentage (%)
Assessment indicator system for disease activity and severity in ulcerative colitis	Partial Mayo Score	23	2.41
	Full Mayo Score	107	11.2
	Modified Mayo Score	116	12.15
	Stool Frequency Score	176	18.43
	Rectal Bleeding Score	188	19.69
	Mayo Endoscopic Score	218	22.83
	Physician Global Assessment	60	6.28
	Simple Clinical Colitis Activity Index	33	3.46
	Truelove-Witts classification	4	0.42
	Pediatric Ulcerative Colitis Activity Index	13	1.36
	Urgency Numeric Rating Scale	1	0.10
	Montreal classification for ulcerative colitis severity	1	0.10
	Ulcerative Colitis Endoscopic Index of Severity	6	0.63
	Robarts Histopathology Index	4	0.42
	Modified Truelove-Witts Severity Index	1	0.10
	Histological remission	4	0.42
Biomarkers, inflammatory laboratory indicators, and immune/pharmacokinetic-related assessments in ulcerative colitis	Hydrogen Sulfide	2	1.71
	Fecal calprotectin	21	17.95
	C-reactive protein	15	12.82
	Erythrocyte sedimentation rate	7	5.98
	Serum cytokines	13	11.11
	Colonic NF-κB protein	2	1.71
	White blood cells	1	0.85
	Human leukocyte antigen-DR high (HLA-DRhi)	1	0.85
	Fluorescence cell detection	1	0.85
	Physical and chemical examination	33	28.21
	Fibroblast products	1	0.85
	CTLA-4 expression	1	0.85
	Pharmacokinetic/pharmacodynamic (PK/PD) indicators	19	16.24
Safety and adverse events	–	96	100
Nutrition and diet assessment indicators in ulcerative colitis	Body Mass Index	1	14.29
	Nutritional Risk Screening	2	28.57
	Dietary habits questionnaire	1	14.29
	Skinfold thickness	1	14.29
	14-item Mediterranean Diet Adherence Screener (14-MEDAS)	1	14.29
	Broccoli Dietary Questionnaire	1	14.29
Quality of life and symptom evaluation indicators in ulcerative colitis	Health-related quality of life (HRQOL) measured using the Short Inflammatory Bowel Disease Questionnaire (SIBDQ)	20	66.67
	Health questionnaire	2	6.67
	Clinical Global Impression-Improvement scale (CGI-I)	2	6.67
	Bristol Stool Form Scale (BSFS)	2	6.67
	Gastrointestinal Symptom Rating Scale (GSRS)	1	3.33
	Short Form 36 Health Survey (SF-36)	3	10.00
Treatment outcomes and clinical endpoint indicators in ulcerative colitis	–	60	100
Microbiome and molecular pathology indicators in ulcerative colitis	Microbiome analysis	14	73.68
	Pathological examination	2	10.53
	Gene expression	3	15.79
Symptom scoring and clinical manifestation indicators in ulcerative colitis	–	9	100
Inclusion and exclusion status of the overall trial data	–	3	100

#### 3.6.2 Secondary outcome measures

Among the secondary outcome measures, the system was characterized primarily by the Mayo score, supplemented by laboratory tests and safety events. The Mayo scoring system again accounted for the vast majority. Among them, FMS was used in 133 studies, representing 23.92%, making it the most commonly used of the three Mayo scoring systems. MMS ranked second, with 126 studies accounting for 22.66%. PMS was comparatively less common, with 70 studies accounting for 12.59%. Among laboratory indicators, fecal calprotectin (137 studies, 25.51%), C-reactive protein (107 studies, 19.93%), and erythrocyte sedimentation rate (42 studies, 7.82%) remained the three most frequently reported laboratory tests, and they were even more common than in the primary outcome measures. In addition, serum cytokines (48 studies, 8.94%) and pharmacokinetic/metabolic assessments (72 studies, 13.41%) were more frequently reported than in the primary outcome measures. This reflects a preference for quantitative biomarkers that are noninvasive, reproducible, and highly correlated with the severity of intestinal lesions.

The Geboes score was the most commonly used endoscopic scoring tool, appearing in 76 studies (50.00%). Quality-of-life measures represented by IBDQ/SIBDQ appeared 120 times (63.16%), indicating that this evaluation metric has been widely incorporated into the assessment system of clinical trials for active UC. However, the proportion of patient daily-experience measures such as the bowel urgency numerical rating scale (18 studies, 31.03%) and the stool consistency scale (3 studies, 5.17%) was relatively low, suggesting that there is still room for further improvement in the development and exploration of patient-reported outcomes. Integrating patient-reported outcomes with physician-driven scoring systems and incorporating both into the evaluation framework is key to ensuring that trial design is both robust and scientifically sound.

Adverse events/safety, as a secondary outcome, appeared in 152 clinical trials, and the high frequency of trial outcome observation (143 studies) suggests that safety evaluation in clinical trials of active CU has shifted toward avoiding corticosteroids and reducing the risk of surgery. The treatment trend has shifted from controlling and improving symptoms to altering the natural course of the disease. Traditional Chinese medicine comprehensive symptom-syndrome scoring appeared 21 times (95.45%), while treatment-related sensations after acupuncture were reported only once (1 study, 4.55%), indicating low frequency and limited use. Characteristics of the secondary outcome measures are presented in **Table 5**.

**Table 5:** Secondary outcome measures of active ulcerative colitis research.

Characteristics (Secondary evaluation index)	Category	Included literature	Record percentage (%)
Assessment indicators for disease activity in ulcerative colitis	Full Mayo Score	133	23.92
	Partial Mayo Score	70	12.59
	Modified Mayo Score	126	22.66
	Ulcerative Colitis Disease Activity Index	6	1.08
	Simple Clinical Colitis Activity Index	29	5.22
	Pediatric Ulcerative Colitis Activity Index	19	3.42
	Truelove-Witts classification	6	1.08
	Lichtiger Index	12	2.16
	Rachmilewitz Endoscopic Index/Ulcerative Colitis Endoscopic Severity Index	25	4.5
	Clinical Global Impression/Patient Global Impression	1	0.18
	Stool Frequency Score	21	3.78
	Rectal Bleeding Score	21	3.78
	Mayo Endoscopic Score	85	15.29
	Physician Global Assessment	2	0.36
Inflammatory biomarkers and pharmacokinetic indicators in ulcerative colitis	Fecal calprotectin	137	25.51
	C-reactive protein	107	19.93
	Erythrocyte sedimentation rate	42	7.82
	Serum cytokines	48	8.94
	Platelets	9	1.68
	White blood cells	18	3.35
	Albumin	15	2.79
	Other laboratory tests	89	16.57
	Pharmacokinetic/pharmacodynamic (PK/PD) indicators	72	13.41
Safety and adverse events	--	152	100
Nutrition, lifestyle, and physical status–related indicators in ulcerative colitis	Basic vital signs	26	70.27
	Dietary adherence	7	18.92
	Changes in nutritional status Patient-Generated Subjective Global Assessment (PG-SGA)	1	2.7
	International Physical Activity Questionnaire-Short Form (IPAQ-SF)	3	8.11
Symptom outcomes in ulcerative colitis	Urgency Numerical Rating Scale (UNRS/NRS)	18	31.03
	Bristol Stool Form Scale (BSFS)	3	5.17
	Single-symptom diary	37	63.79
Treatment outcome indicators in ulcerative colitis	Steroid remission/Whether surgery is needed/Disease recurrence	51	35.66
	Treatment remission	92	64.34
Histopathological scoring in ulcerative colitis	Geboes score	76	50
	Robarts Histopathology Index (RHI)	24	15.79
	Nancy Histological Index (NHI)	15	9.87
	Modified Riley Score (MRS)	1	0.66
	Histological remission	33	21.71
	Intestinal barrier function	3	1.97
Microbiota indicators in ulcerative colitis	Fecal microbiota/fecal metabolomics/fecal pH/microbial colonization/sulfate-reducing bacteria	60	85.71
	Intestinal ultrasound	1	1.43
	Cellomics-mitochondria	2	2.86
	Gene expression	6	8.57
	Viral load detection	1	1.43
Patient-reported outcomes (PROs), quality of life, psychological status, and symptom-related assessment indicators in ulcerative colitis	Inflammatory Bowel Disease Questionnaire (IBDQ-32)/Short IBDQ (SIBDQ) — health-related quality of life (HRQOL) measured using the short inflammatory bowel disease questionnaire	120	63.16
	SF-36/SF-12/EQ-5D-5L/EQ-5D-3L	22	11.58
	Functional Assessment of Chronic Illness Therapy–Fatigue (FACIT-F)	17	8.95
	Pittsburgh Sleep Quality Index (PSQI)/Insomnia Severity Index (ISI)	3	1.58
	Hospital Anxiety and Depression Scale (HADS)/PHQ-9/Beck Depression Inventory-II (BDI-II)/Beck Anxiety Inventory (BAI)	14	7.37
	Gastrointestinal Symptom Rating Scale (GSRS)	4	2.11
	Work Productivity and Activity Impairment Questionnaire (WPAI-IBD)	4	2.11
	Patient-Doctor Relationship Questionnaire (PDRQ-9)	2	1.05
	Visual Analog Scale (VAS)	4	2.11
Traditional Chinese medicine indicators in ulcerative colitis	Post-treatment Acupuncture Sensation Scale (MASS)	1	4.55
	Traditional Chinese Medicine (TCM) Composite Symptom and Syndrome Score	21	95.45

## 4 Discussions

This study reviews the current status of clinical trials in active UC, providing a reference for researchers to better understand trial design for this disease. Previous studies have mainly focused on pathogenesis and drug therapy (23, 24). However, there has been a lack of scoping reviews specifically addressing clinical trials in active UC. This is the first scoping review of the characteristics of clinical trials related to active UC.

Over the past decade, the number of registered clinical trials and publications has shown a gradual upward trend, with a particularly pronounced increase after 2019. This suggests that research on clinical trials related to active ulcerative colitis has entered a period of high interest.

In clinical research, biologics and small-molecule targeted therapies have become the core focus of current clinical studies in active ulcerative colitis. The data show that biologic-related studies accounted for 116 trials (21.25%), small-molecule targeted drugs for 77 trials (14.10%), whereas traditional drug therapies (aminosalicylates, corticosteroids, and conventional immunosuppressants) accounted for only 32 trials (5.86%). This indicates that the research direction is gradually shifting from broad anti-inflammatory treatment toward precision therapy targeting specific molecular pathways. Among biologics, anti-TNF-α agents (29 studies, 5.56%) and emerging target-specific biologics (32 studies, 6.13%) were included. Anti-TNF-α agents still maintained relatively high research activity, and both emerging target-specific biologics and small-molecule targeted therapies remained at a high level of study activity (25). This trend is consistent with recent advances in biomarkers and therapeutic targets for ulcerative colitis. It has been suggested that combination biologic therapy and the development of novel receptor inhibitors may represent a more promising direction for ulcerative colitis treatment (26). However, the use of biologics still faces several challenges, such as patient resistance and drug tolerance, as well as the lack of clear predictive biomarkers for biologic response (27). Few trials in this review reported biomarker-based stratification or directly compared combination regimens with monotherapy, representing gaps in the current trial landscape. In addition, given the potential additive immunosuppressive effects of combination treatment (28), long-term safety monitoring was infrequently reported among included trials.

Among the interventions, one research direction involves drug repurposing, in which medications originally approved for other indications are used in new therapeutic contexts. These pharmacological interventions reflect drug repurposing and illustrate a shift from a “disease-based” approach to a “mechanism-based” approach in drug selection. However, the mechanisms and comparative effectiveness of these agents were not assessed in this review.

Among all 522 included studies, supportive nursing interventions (23 studies, 4.21%), cellular and blood-derived interventions (17 studies, 3.11%), neuromodulation therapies (6 studies, 1.10%), and hyperbaric oxygen therapy (5 studies, 0.92%) accounted for relatively small proportions. However, as emerging research directions, these interventions have shown a marked increase over the past five years. Comparative analysis showed that the number of related studies increased by 54.55%, from 33 studies during 2016–2021 to 51 studies during 2021-2026. The main intervention types included dietary guidance, mesenchymal stem cell therapy, TLA intestinal leukocytapheresis, hyperbaric oxygen therapy, and sacral nerve stimulation. Taking mesenchymal stem cell therapy (MSCs) as an example, its effectiveness in treating UC has been demonstrated, and it offers the advantage of regenerative capacity. However, the proliferative and differentiation potential of MSCs may also increase the risk of tumorigenesis, which may be one reason why this type of therapy accounts for a relatively small proportion of current interventions (29). Dietary guidance, another broadly defined intervention, was also common in the included clinical trials. However, apart from Mediterranean diet, semivegetarian diet, and plant-based high-fiber diet, most studies did not clearly specify the exact dietary pattern, instead using vague descriptions such as “dietary guidance” or “dietary intervention.” Recent research suggests that not all dietary interventions achieve the expected effects. Dietary interventions were evaluated in a small proportion of included trials, and the specific dietary patterns studied varied. Most trials did not clearly specify the dietary pattern, instead using broad terms such as “dietary guidance”. This variability in intervention description limits comparability across trials.

Regarding the distribution of the four subscores, MES can be seen as not only the most diagnostically valuable assessment tool, but also one with strong persuasive power. The central role of MES is consistent with the current trend in clinical trials of active ulcerative colitis, where endoscopic remission is commonly used as the primary endpoint (218 studies, 41.76%). However, MES has relatively low sensitivity to disease change and may not adequately characterize varying degrees of intestinal injury, such as erosion, ulceration, or extensive mucosal inflammation (30). The AGA 2024 clinical practice update, while affirming the clinical value of endoscopic scoring systems, also pointed out limitations such as subjectivity and incomplete validation in some tools; it further listed Ulcerative Colitis Endoscopic Index of Severity (UCEIS) alongside MES as a commonly used instrument, suggesting that UCEIS may serve as a useful complement to or substitute for MES (31). RBS and SFS are patient-reported outcomes that can fully reflect patients’ symptom burden. In contrast, PGA is a clinician’s overall judgment based on objective information. The lowest use of PGA may be related to high inter-observer variability caused by subjective physician judgment.

Among the disease characteristics of the included samples, 25 studies (4.79%) did not clearly define the severity of disease in the enrolled patients, which to some extent weakened baseline comparability across studies. Using the Mayo scoring system or other scoring systems as standardized tools not only provides a clear quantitative basis for baseline characteristics of the enrolled population, but also highlights significant differences in disease severity, treatment urgency, and complications between mild-to-moderate and moderate-to-severe UC. Such explicit patient stratification is an important methodological consideration.

This review also describes the widespread presence of small-scale studies and short-term follow-up in current clinical trials. 65.13% of trials enrolled fewer than 100 participants, and only 9.58% had sample sizes greater than 300. 12–24 weeks (136 studies, 26.05%) and 24 weeks or longer (117 studies, 22.41%) were predominant, and 12 weeks (96 studies, 18.39%) was the most common intervention duration. This suggests that 12 weeks is a critical time point for assessing efficacy. The current distribution of intervention duration is dominated by short-term studies, with long-term studies being relatively sparse. Existing evidence mainly reflects short-term treatment effects, while assessment of long-term efficacy and safety remains limited. In particular, short follow-up is one of the major issues that must be addressed. Most patients with UC relapse within 2–3 years after remission (32), but overly short follow-up periods may lead to overestimation of efficacy and underestimation of risk, and are insufficient to evaluate the long-term benefits of histologic remission. Compared with other methodological limitations, clinical studies on active ulcerative colitis show relatively high completeness and inclusiveness in reporting sex. To explore the relationship between disease severity and sample size in active ulcerative colitis, this study summarized the distribution of disease severity across different sample-size strata. In the 0–50 sample-size range, there were 76 studies of mild-to-moderate UC (14.56%) and 87 studies of moderate-to-severe UC (16.67%), both accounting for large proportions, with the latter slightly more common. In the 51–100 sample-size range, studies of mild-to-moderate UC increased significantly to 65 (12.45%), becoming the main component of that range. In contrast, the number of moderate-to-severe studies in the 51–100 range decreased to 37 (7.09%). In all subsequent sample-size ranges of ≥101, the proportion of moderate-to-severe studies exceeded that of mild-to-moderate studies, and this advantage expanded with increasing sample size. Overall, sample sizes for mild-to-moderate UC were mostly below 150, whereas sample sizes for moderate-to-severe UC were mostly below 250. These distributional characteristics can inform the design of future clinical trials or observational studies by helping researchers plan appropriate sample-size ranges according to disease severity, thereby improving feasibility and persuasiveness. In summary, current clinical studies on active ulcerative colitis are affected by various methodological deficiencies. Overall, the reported methodological features of clinical trials for active UC suggest areas for improvement, especially because of insufficient sample sizes and short follow-up periods. These limitations not only affect the accuracy of efficacy evaluation and may overestimate long-term effectiveness, but also lead to incomplete characterization of late events and adverse-event profiles. These findings suggest that large-scale, long-duration trials with extended follow-up remain uncommon in this field. The distribution of disease severity by sample size group is shown in **Table 6**.

**Table 6:** Distribution of disease severity by sample size group.

Sample size	Counts and percentages (%)							
	Mild-to- moderate	Moderate-to -severe	Mild-to-severe	Mild	Moderate	Severe	Unclear	Total
0 ≤ Sample size ≤ 50	76 (14.56)	87 (16.67)	10 (1.92)	3 (0.57)	10 (1.92)	10 (1.92)	11 (2.11)	207 (39.66)
51 ≤ Sample size ≤ 100	65 (12.45)	37 (7.09)	5 (0.96)	3 (0.57)	10 (1.92)	6 (1.15)	7 (1.34)	133 (25.48)
101 ≤ Sample size ≤ 150	19 (3.64)	32 (6.13)	1 (0.19)	1 (0.19)	4 (0.77)	5 (0.96)	5 (0.96)	67 (12.84)
151 ≤ Sample size ≤ 200	4 (0.77)	19 (3.64)	1 (0.19)	0	2 (0.38)	0	0	26 (4.98)
201 ≤ Sample size ≤ 250	8 (1.53)	17 (3.26)	1 (0.19)	1 (0.19)	2 (0.38)	0	0	29 (5.56)
251 ≤ Sample size ≤ 300	2 (0.38)	7 (1.34)	0	0	1 (0.19)	0	0	10 (1.92)
301 ≤ Sample size ≤ 350	2 (0.38)	6 (1.15)	0	0	0	0	0	8 (1.53)
351 ≤ Sample size ≤ 400	1 (0.19)	7 (1.34)	0	0	0	0	0	8 (1.53)
401 ≤ Sample size ≤ 450	1 (0.19)	3 (0.57)	0	0	0	0	0	4 (0.77)
Sample size ≥451	2 (0.38)	25 (4.79)	0	0	1 (0.19)	0	2 (0.38)	30 (5.75)
Total	180 (34.48)	240 (45.98)	18 (3.45)	8 (1.53)	30 (5.75)	21 (4.02)	25 (4.79)	522

At present, the selection of outcome measures in clinical trials for active UC shows structural imbalance. This is mainly reflected in the singularity of chosen endpoints, limited use of novel scoring tools, short observation periods for long-term safety, and a lack of patient-centered indicators. Among primary outcomes, the Mayo scoring system dominates overwhelmingly (246 studies, 47.13%). Within the Mayo system, MMS was most common (116 studies, 22.22%), followed by FMS (107 studies, 20.50%). Among the subitems covered by the various Mayo score versions, MES is the only indicator that directly reflects mucosal status in the intestine (33). Given that mucosal healing has been established as a long-term goal of UC treatment (34), MES, as the most frequently reported indicator for assessing endoscopic healing, has gained broad acceptance among researchers, and this high level of acceptance explains its statistical dominance. By contrast, PGA is a composite clinical judgment integrating symptoms, signs, laboratory tests, and other dimensions. Although it offers the advantage of rapid assessment, it does not accurately capture mucosal healing and is subject to substantial interphysician variability; therefore, it was removed from the MMS. SFS and RBS reflect patients’ actual symptom burden and are core indicators of subjective disease experience, so they remain common components of the Mayo score. It is worth noting that there is a pronounced imbalance in the dimensional distribution of assessment measures. Although safety outcomes account for a certain proportion (18.39%), they are often limited to rough recording of adverse events and lack standardized long-term safety monitoring. In addition, there is a significant difference in the frequency of IBDQ/SIBDQ as primary outcomes (20 studies) versus secondary outcomes (120 studies), indicating their low priority but high acceptance. Nutritional status and diet-related assessment measures are even less common. This suggests that current clinical trials still focus mainly on traditional clinical disease-activity endpoints such as the Mayo scoring system, while paying insufficient attention to patient-reported experience and functional status. Among laboratory measures, unlike serum markers such as CRP, fecal calprotectin is derived from neutrophils in the intestinal mucosa and can specifically reflect the degree of intestinal inflammation without interference from inflammation elsewhere in the body. This feature has made fecal calprotectin the most widely used biomarker in active ulcerative colitis research (35). In addition, 57 studies (10.92%) did not include any secondary outcome measures at all, reflecting a systematic neglect of comprehensive efficacy evaluation. UCEIS was rarely used in the included trials. Similarly, patient-reported outcomes were infrequently used as primary endpoints, long-term safety monitoring was inconsistently reported, and a standardized core outcome set covering multiple assessment dimensions was lacking. These gaps in outcome measure selection represent areas that future research may address.

Similarly, histopathological scoring is increasingly recognized as an adjunct treatment target. The Geboes score (36) was the most frequently used histopathological scoring (76 studies), followed by the Robarts Histopathology Index (37) (24 studies) and the Nancy Histological Index (38) (15 studies), all of which show good concordance in distinguishing histological remission from activity. Pediatric trials were uncommon: only 19 studies (3.64%) specifically enrolled children, and PUCAI was used as a primary outcome in only 13 studies. This suggests that pediatric populations remain under-represented in current trial design.

Across all clinical trials, 80.65% used randomization, reflecting attention to standard methodological practices. The frequent use of open-label designs in clinical research may be due to investigators’ concern for operational convenience and the difficulty of blinding certain interventions. The number of quadruple-blind designs was much higher than that of triple-blind and single-blind designs, indicating that higher-quality trials with more rigorous reported blinding procedures have begun to include data analysts and other personnel in blinding procedures in order to further reduce assessment bias. The distribution of trial-arm numbers suggests that two-arm designs are the mainstream in current active ulcerative colitis studies, while multi-arm designs indicate a trend toward greater precision and comprehensiveness in research.

Notably, 58.43% of included trials did not clearly report the follow-up duration. This is a major methodological concern, as insufficient reporting of follow-up limits the assessment of long-term efficacy and safety, and hinders comparability across trials.

Current clinical research still faces various methodological problems, including insufficient blinding and limitations in the comparability of control groups in two-arm trials. These issues may introduce detection bias and affect the objectivity of the final results. These methodological issues were commonly observed across the included trials. Enhancing the implementation of blinding, encouraging multi-arm trials, and improving the completeness and verifiability of registration information are important directions for future research design.

Taken together, the included trials showed substantial heterogeneity in study populations, intervention categories, trial design, and outcome measure selection. This heterogeneity limits direct comparison across trials and hinders evidence synthesis. Key gaps identified in this review include the infrequent use of UCEIS, histological remission, and patient-reported outcomes as primary endpoints; the low proportion of pediatric-specific trials; and the inconsistent reporting of follow-up duration. These gaps may inform the design of future clinical trials in active UC.

First, consistent with scoping review methodology, no formal critical appraisal or risk of bias assessment was performed. Therefore, the findings should be interpreted as a descriptive mapping of the current landscape of clinical trial design rather than as a quality-graded evidence synthesis. Second, no quantitative evidence synthesis or meta-analysis was conducted, as the primary objective was to characterize the breadth of trial design and outcome measures rather than to estimate pooled treatment effects. Third, potential publication bias may exist, as grey literature, conference abstracts, and unpublished trial data were not systematically searched. Fourth, although a comprehensive search was conducted across three major databases, some relevant trials may have been missed due to limitations in search terms, database coverage, or indexing. Fifth, the restriction to records registered or published within a specific 10-year window may have excluded older but still relevant trial designs. These limitations should be considered when interpreting the findings, and future systematic reviews with formal quality assessment and evidence synthesis may be warranted for specific research questions identified by this scoping review.

## 5 Limitations of the study

This scoping review identified several limitations in the design of current clinical trials for active ulcerative colitis. First, the included trials showed substantial heterogeneity in study populations, intervention categories, trial design, and outcome measure selection, which limits direct comparison across trials and hinders evidence synthesis. Second, many trials were small in scale. Among the 522 included studies, 65.13% enrolled fewer than 100 participants, and only 9.58% had sample sizes greater than 300. Third, follow-up duration was frequently insufficient or incompletely reported. As many as 305 studies (58.43%) did not clearly report the follow-up period, and long-term follow-up of more than 12 months was reported in only 22 studies (4.21%). Fourth, intervention duration was dominated by short- and medium-term designs. Although 12–24 weeks (136 studies, 26.05%) and 24 weeks or longer (117 studies, 22.41%) were the most common, 220 studies (42.15%) had intervention periods of less than 12 weeks. Fifth, methodological reporting was incomplete in some trials. Twenty-one studies (4.02%) did not clearly report blinding design, 49 studies (9.39%) did not clearly report intervention duration, and 25 studies (4.79%) did not clearly specify the severity of active ulcerative colitis. Sixth, outcome measures were structurally imbalanced. The Mayo scoring system dominated primary outcomes, while UCEIS was rarely used, patient-reported outcomes were infrequently used as primary endpoints, and long-term safety monitoring was inconsistently reported. Seventh, pediatric populations were under-represented. Seventy-six studies (14.56%) allowed participants younger than 18 years in their age eligibility criteria, and PUCAI was used as a primary outcome in only 13 studies. Finally, few trials reported directly compared combination regimens with monotherapy, and the mechanisms and comparative effectiveness of repurposed agents were not assessed in this review. These limitations should be considered when interpreting the findings and should inform the design of future trials in active UC.

## 6 Conclusion

This scoping review summarizes the current landscape of clinical trials for active ulcerative colitis. Trial numbers increased after 2019, with biologics (116 studies, 21.25%) and small-molecule targeted drugs (77 studies, 14.10%) as the core interventions. Randomized controlled trials predominated (420 studies, 80.46%), but 65.13% of studies enrolled fewer than 100 participants and 58.43% of studies did not clearly report follow-up duration.

Outcome measures were dominated by the Mayo scoring system, while UCEIS, histological remission, and patient-reported outcomes were infrequently used as primary endpoints. Substantial heterogeneity was observed in study populations, interventions, trial design, and outcome measures. Future trials should prioritize large-scale, long-term studies with standardized, comprehensive, and patient-centered outcome measures.

## Data Availability

The original contributions presented in the study are included in the article/**Supplementary Material**. Further inquiries can be directed to the corresponding authors.
