# Supplementary material for: Design and outcome measures of clinical trials for active ulcerative colitis: a scoping review based on databases
Source: Front Immunol. 2026 Oct 19;17:1932604. doi: 10.3389/fimmu.2026.1932604 (PMC13724425; doi:10.3389/fimmu.2026.1932604)
Supplement: Supplementary file 1 [file Table2.docx]

**Supplementary Table 2 Search strategy**

| **The search strategy for PubMed** | |
| --- | --- |
| **Number** | **Search terms** |
| #1 | ((((Colitis Gravis[Title/Abstract]) OR (Idiopathic Proctocolitis[Title/Abstract])) OR (Inflammatory Bowel Disease[Title/Abstract])) OR (Ulcerative Colitis Type[Title/Abstract])) OR (Ulcerative Colitis[Title/Abstract]) |
| #2 | (clinical trials OR randomized controlled trials) |
| #3 | #1 AND #2 |

**The search strategy for** **Clinicaltrials**

(Colitis Gravis OR Idiopathic Proctocolitis OR Inflammatory Bowel Disease OR Ulcerative Colitis Type OR Ulcerative Colitis)

**The search strategy for ChiCTR**

(Colitis Gravis OR Idiopathic Proctocolitis OR Inflammatory Bowel Disease OR Ulcerative Colitis Type OR Ulcerative Colitis)
